# The Relative Death-Rates from Cancer and Consumption1Read before the Alumni Association of the Medical Department of the University of Buffalo, April 25, 1899.

**Published:** 1899-07

**Authors:** John H. Pryor

**Affiliations:** Buffalo, N. Y.


### THE RELATIVE DEATH-RATES FROM CANCER AND CONSUMPTION.[1]

By JOHN H. PRYOR, M. D., Buffalo, N. Y.

It is a part of probability that many improbable things will happen.

—ARISTOTLE.

THE term consumption when employed in this paper, refers to pulmonary tuberculosis only.

Recently considerable literature has appeared in medical journals, periodicals, and the daily newspapers concerning the death-rate from cancer in this state. It has been compared with consumption to show a relative gain which has surprised the profession and the public. Wild statements have been accepted without examination, and they have caused alarm and given rise to opinions which are

1. Read before the Alumni Association of the Medical Department of the University of Buffalo, April 25, 1899.

erroneous and misleading. One writer of eminence has repeatedly published statistical information, conclusions and predictions with reference to the death-rate from cancer which are startling if true.

At this time special attention will be invited to two recent contributions. (1) An article entitled, A further study into the frequency and nature of cancer, *Medical News*, April 1, 1899, and, (2) First annual report of the director of the New York State Pathological Laboratory of the University of Buffalo, transmitted to the legislature January 26, 1899. This report to the legislature is an official communication, and the following quotation explains the relations of the institution and the state : "Last year the legislature appropriated $10,000 to the Medical Department of the University of Buffalo for the purpose of equipping and maintaining a laboratory, to be devoted to the study of the causes, mortality rate and treatment of cancer." In each article figures derived from the reports of the state board of health are given, and the increase of cancer for the last eleven years is offered in support of this remarkable conclusion.

"A careful study of these tables permits one to make the following startling prophecy : If during the next ten years the relative death-rates are maintained we shall find that ten years from now, viz.: 1909, there will be more deaths in New York State from cancer than from consumption, small-pox and typhoid fever combined." The extraordinary nature of this prediction is appreciated and italics are employed to emphasize its portentous meaning. Incredulity has led to a scrutiny of the data presented in proof of this assertion.

In the *Medical News* the following figures are published to show the gain in mortality from cancer and consumption for the last eleven years inclusive. "Deaths from cancer in New York State, 1887, 2,363; consumption, 11,609. In 1898 there were 4,456 deaths from cancer and only 12,552 from consumption."

The figures for 1898 are incorrect,—they should be, deaths from cancer, 4,385; death from consumption, 12,979—a decrease of 71 for cancer and an increase of 427 for consumption. Now to estimate the increase from 1887 to 1898 without any reference to yearly increase or averages is manifestly inaccurate, but allowing that privilege, the gain in the death-rate of cancer is 85 per cent. and consumption, 11.8 per cent.; if fractions of percentages be dropped the gain of cancer over consumption is 77 per cent. for eleven years or twelve years inclusive.

The sources of error due to the peculiarities displayed by disease and influencing conditions are so numerous and glaringly apparent, that one can place slight credence upon any estimate for future years. But if any one has sufficient interest to compute the probable increase in deaths from cancer, according to the percentage given he will find that cancer must increase three and a half to four times faster in the next ten years than it has in the last eleven years to destroy as many lives as consumption. "If the relative death-rates are maintained" cancer will destroy as many as consumption and typhoid fever in from thirty-five to forty years or about 1945. I repeat, all such generalisations and predictions are unjustifiable scientifically, and only excite morbid interest and promote novel theories which mislead.

If any consideration of typhoid fever and small-pox be eliminated, some confusion and misapprehension may be avoided. The dread disease small-pox caused one death in this state last year, and the very small percentage of deaths from typhoid fever, in proportion to the number who suffer from that disease, may lead to false views of its prevalence as compared with cancer. Perhaps the danger of any prognostication regarding the future conduct of a disease may be illustrated in passing by citing the death-rate from typhoid fever last year when it jumped up 469 over 1897, due largely to epidemics in military camps. Consumption increased its death-rate 338, and cancer 254, a gain of 503, which cancer must overcome at its average increase of 160 per year. In the report to the legislature mentioned, tabulated figures prepared from the reports of the state board of health are furnished and the principal part of the report devoted to mortality is employed to show the relative increase of cancer and decrease of consumption. Statistical table 6, page 15, contains the estimated population for each death from these two diseases from 1887 to 1898. The deductions are based upon the whole increase again, not upon the average. To quote: "Thus in 1887 one person in every 2,402 died of cancer, and one of every 499 of consumption." "The table also shows an increase (of deaths from cancer) of 40 per cent. during the eleven years tabulated." "In other words, in 1887 one out of every 2,400 died of cancer, in 1898 one out of every 1,500." "The same table shows that the mortality rate from consumption has shown a marked decrease." The figures given for 1898 are 1 of 1,568 for cancer and one of 544 for consumption. They are incorrect, and should be 1,594 and 540 respectively, a decrease of twenty-six for cancer and an increase of four for consumption. A consideration of these figures given in the statistical table leads the author to the alarming prediction, in italics, intended to show that the deaths from cancer will exceed those of the three diseases already mentioned.

It will be found that an increase of 40 per cent. for the next ten years, even provided the death-rate from consumption remains the same, will yield the following result: Deaths from cancer, 6,139; deaths from consumption, 13,000.

An estimate of the average death-rate to the population to equalise variations show for eleven years, cancer one death for 2,210, and consumption one death for 476 population. Thus consumption exhibits an average death-rate per year much higher than the figures given in the summary above. Once more the only safe conclusion possible is that cancer must increase three and a half to four times faster in the next ten years than during the last eleven to be abreast of consumption as a cause of death.

The relative percentage of all deaths or the number per 1,000 deaths give the same result: Consumption causes about 11 per cent. of all deaths. The percentage for each of the eleven years wavers from 10 6.10 per cent. to 11 per cent. The deaths from cancer show an average gain of 1 per cent—an increase of 1 5.10 per cent. in 1898 over 1887. The number of deaths from cancer in 1898 is 3 7.10 per cent. of total deaths for that year. The average increase of deaths from cancer for eleven years is 160 each year, and at that rate of increase for ten years the total number of deaths from that disease in 1909 would be 5,985. Consumption increased its number of deaths on an average of 136 per year, and if the same ratio is maintained 14,839 will die of that disease in 1909.

This computation is introduced simply to illustrate a discrepancy in estimating results based upon vital statistics and to show that the expected increase of cancer is unwarrantably exaggerated. It may be claimed that a "marked decrease" for consumption is not revealed. The alleged decrease of consumption in this state is due largely to gross exaggeration, and somewhat to sophistical misuse of statistics selected for a special purpose. Nor can it be fairly claimed that the death-rate from cancer increases progressively. By dividing a series of twelve years into two periods of six years and estimating the increase, it will be found to be very slight, and certain districts of the state show a tendency to preserve an unchanging death-rate or a gradual decrease in rapidity of increase. The reports from New York City for thirty-one years, divided into three decades, reveal an increase represented by 55 per cent., 73 per cent., 39 per cent., and for the last five years 23 per cent. Cancer is not the only disease characterised by a steadily increasing death-rate. Diseases of the urinary system and particularly Bright's disease and pneumonia have increased almost if not quite as fast, especially if considered in relation to mortality and the number affected. The fact that cancer is increasing is best illustrated by the advance in percentage of the death-rate in relation to the general death-rate. However grave the outlook may be, there is no justification for the expected increase of more than 300 per cent. in ten years, when by the most liberal and questionable methods the past gain has been 85 per cent. in eleven years.

For some reason not explained by an examination of the mortality returns, the statement has repeatedly been made that a certain locality in New York State, of which Buffalo has been said to be the center, belonged to a distinct "cancer-belt," where cancer was prevalent to a remarkable degree. The belief in this myth has become quite wide-spread and recently inquiries from a distance have been reaching physicians here. There seems to be no reason why the rumor should go longer without a denial. It is doing the City of Buffalo no particular good, especially in view of its boasted low death-rate. The returns of deaths from cancer in the Lake Ontario and western district, in which Buffalo is included, gives a death-rate of sixty-one per 100,000 for 1896 and 1898. This shows not only no increase per population, but a rate below the average for the state. The death-rate for Buffalo is 58 per 100,000 or below that of the district. It is also below that of other cities in different parts of the state. Compared with the State of Massachusetts, certainly far removed from any supposed "cancer-belt," and noted for the reliability of its vital statistics, it is found that the rate for that state in 1895 was 64 per 100,000 or 6 per 100,000 more than Buffalo. In Massachusetts the death-rate from cancer has almost trebled in forty years and in Michigan is about three times as great as it was thirty-one years ago. In both states the increase is somewhat greater than in New York State in spite of the larger population.

There are possible sources of error which must be considered as bearing upon the death-rate and prevalence of cancer and consumption. The increase in reports of death from cancer may be accounted for to an indefinite extent by improvements in diagnosis and added knowledge due to operative interference, particularly in the abdominal cavity. We must also bear in mind that an unknown number of patients come to large medical centers in this state and those dying add to the mortuary records. Practically every case of cancer is reported, because almost invariably fatal. On the other hand, many consumptives leave the state for other climates and the deaths are unrecorded here. A certain unknown percentage recover and at least 25 per cent. of autopsies reveal evidences of tuberculosis not fatal or healed. The difficulties in securing sick, industrial and life insurance and efforts to protect the family at times, are giving rise to evasion and false returns.

Surgical treatment of tuberculosis, which has improved remarkably in recent years has lessened the number of consumptives by the eradication of foci of infection. Finally, the fact cannot be emphasized too strongly that consumption indicates an affection of one organ and tuberculosis of all other organs is not included in recent comparative death-rates. This is partial and misleading. If all the deaths from tuberculosis in the unclassified list and the deaths in childhood due to the same cause were added, the disparity between the deaths from cancer and consumption already shown would be still further magnified.

Of late the assertion that consumption has materially decreased and steadily declined in New York State has been reiterated without any explanatory data of a broad or comprehensive character. It is now sixteen years since Koch's discovery of the cause of tuberculosis, and since new methods of prevention were suggested. The death-rate continued to advance numerically until 1887, then fluctuated until 1892, since which time it has remained stationary or has slightly declined. Since 1887 the average yearly increase has been 136 for twelve years. In 1897 there was a marked decrease and the enthusiasts made promising conjectures and ascribed great results to so-called prevention. In 1898 there was an increase of 338 and 12,979 deaths—very near the 13,000 average again. Whenever a decrease has occurred it has not been general, and the slight diminution in recent years is largely due to a lower death-rate from the diseases in New York City, particularly among females.

In the eight districts of the state, all save the maritime, which includes New York City, the number of deaths per 100,000 population has increased. The percentage of all deaths and the number per 1,000 deaths has remained practically the same except in 1897, and it increased again in 1898. In 1893 the number of deaths per 1,000 of all causes of death was 105.94, average for six years, 108.13, and in 1898, 108.13. Consumption causes as many deaths, but not as many in proportion to the population. The explanation is to be found in the recent great diminution in the general death-rate, and consumption simply shares with other zymotic diseases in the prolongation of life and greater freedom from disease.

It claims its proportion of the small death-rate as it has of the high, but its death-rate does not decrease as rapidly as the general death-rate. The death-rate in New York has decreased 22 per cent. since 1888 and much more rapidly in some cities, viz.: Buffalo, whose death-rate last year was 12.25 per 1,000 and that of the state, 18.10. Compared with a preventable disease, *i. e.*, scarlet fever, the difference is very plain, and with a disease where prevention and saving life are combined, *i. e.*, diphtheria, the remarkable decrease of the latter and the curious constancy of consumption are most striking. To repeat, the number of deaths from all causes in proportion to the population has undergone a most remarkable decrease in the last ten years. The population has steadily increased in this state from 5,800,000 in 1888 to 7,000,000 in 1898, while the death-rate declined. There are fewer deaths from all causes, but consumption continues to claim its proportion. Numerically the death-rate may vary or diminish during certain periods, but tuberculosis in all forms is still responsible for 1.7 of the deaths of this state.

When considered in relation to all the facts, the claim that consumption has markedly decreased is exaggerated, and the statement that the disease has been checked in its spread is more accurate and trustworthy. Furthermore, this limiting of its ravages cannot be ascribed, except in small part, to any special efforts in the way of prevention. The wave of sanitary improvement has probably accomplished the result by removing the cause, increasing vital resistance and eradicating vicious and therefore encouraging environments and conditions.

Modern ideas of prevention have never been put in practice in this state except to a slight extent. Methods which seem to promise much have never been thoroughly or generally tried. What changes this adoption might produce are unknown. Education has not fulfilled the extravagant predictions that consumption would rapidly disappear before its influence after a knowledge of the cause was given to the world. It has aided, but its principal use will be found in making the enforcement of wise precautions easier and more acceptable.

Under existing conditions there seems to be no sure foundation for a belief that consumption will rapidly decline as the chief destroyer of human life. Unless new methods of dealing with the disease are introduced there is no sufficient justification for the pleasing prophecy so often heard, that the scourge will pause in its awful work. That its death-rate will diminish in the next ten years enough to allow cancer to overtake it, obviously calls for no comment.

The fatal disease cancer appears to be increasing with such terrible rapidity that the mere mention of the fact is enough to cause apprehension and alarm and stimulate a desire to learn more of the cause and nature of the malady. Any comparison is unnecessary and is apt to prove harmful by confusing the public mind and distracting attention from problems of the day pressing for solution.

The state has made a commendable move in establishing a state laboratory in which investigations, relating to the cause and nature of cancer may be pursued. If appropriations are liberal enough to allow of a proper prosecution of the work, something may be accomplished. As yet, no proof has been established of its infectious nature or parasitic origin, but many signs point in that direction, and the fact that those qualified to pursue the investigation are pursuing the matter with scientific zeal all over the world makes the future outlook somewhat more hopeful. How near or how far we are from the time when more may be known of cancer cannot at present be conjectured. We know the cause of consumption and the conditions which promote the disease. We know that it is infectious and largely preventable. We know that a percentage, varying from 60 per cent. to 75 per cent. of early cases can be cured and the remainder improved by proper treatment. Therefore an immense and unnecessary loss of life and an appalling and unnecessary amount of suffering prevails in this state because stupid, antiquated, inhuman and wasteful methods of dealing with the consumptive continues.

Broad, general and sensible measures of prevention can be employed without hardship, and the public can thus secure the protection which is its right. Sanitary treatment of the early cases is the humane and ideal method of prevention, but it must be preceded or accompanied by other measures. When the prospect of proper treatment is offered the incipient case, prevention will be hurried and failure to make an early diagnosis will be blameworthy, as it has become in appendicitis. For many indisputable reasons the state must lead the way as other states and countries have done. Until the present crusade against consumption has accomplished something definite, the conditions will remain practically unchanged, and in view of our present knowledge the consumptive will continue to be one of the saddest victims of neglect in the world.

How long the death-rate from consumption will remain a disgrace to the medical profession and the state we cannot know, but the time will not be shortened by the recent tendency to exaggerate the importance of one disease and belittle the dangers from another. The possibility of the death-rate of cancer exceeding that of consumption in 1909 on the present ratio of increase can only be realised by some unlooked for and very unusual occurrence.

Undoubtedly the number of deaths from consumption can be greatly decreased by prevention and proprer care, and if rational methods of relief are ever adapted, the time may come when the wide difference between the mortality rate of consumption and cancer may be greatly diminished or obliterated. At present the supremacy of consumption as the "white scourge of civilisation" is in little danger, and ignorance and false theories will probably help to lengthen the long and necessarily unknown period which must elapse before its new rival will be proclaimed the champion depopulator.

DISCUSSION.

Dr. H. R. Hopkins said it was a subject for congratulation that we have an opportunity occasionally to consider the reasons why the state is interested in this topic. There is nothing for which the medical profession is more celebrated than its conservatism; and perhaps the largest factor in that conservatism is the inertia of the human mind. We have but to refer to the date when Jenner published the results of his twenty years' labor to realise how slow the profession is to take advantage of his discoveries and to estimate how long it will take that profession with the same conservatism and the same inertia to profit by the discoveries of Koch. He thought it was probably not too much to say that the profession as a profession is absolutely unconscious in its methods of treatment of the fact that tuberculosis is a communicable disease. If that end were accomplished in 100 years, he thought we should have done well. Another obstructive element is preconception, and it is very difficult to get out of the head of the old practitioners the idea of its transmissibility by heredity and so long as a vestige of that notion remains our efforts at present will be impotent.

I congratulate the alumni association and the readers of these papers that on this annual gathering so many of us can meet from our several walks of life and notwithstanding our different prejudices, try to carry away something which shall make for the general betterment of the profession. It seems to me we can go to our homes with a clearer conception of the state's interest in the protection of the lives of its citizens, and that it can do something to limit the spread of this communicable disease which spreads, not by heredity, but simply by contact of the susceptible with the infectious principle, which comes from some animal or person infected with tuberculosis.

Dr. DeLancey Rochester—The change of topic has necessarily modified the discussion somewhat. I trust that before it is ended the conductor of the state laboratory (Dr. Gaylord) and others who are interested in these statistics will have an opportunity to present their side of the question. The discussion of the comparative death-rate of cancer and pulmonary tuberculosis is purely a matter of statistics on which I am not prepared to enter. The fact remains, however, that we have an increase in both diseases going on from year to year and the relation of the state to this matter is one of exceeding interest to all. I think the point well taken by Dr. Hopkins, that we as a class are not doing all we can to prevent the spread of tuberculosis. Over and over again people have come to my office suffering from this disease who have been under the care of physicians, yet not one word has been said to them about its infectious nature, and that they themselves can prevent its transmission to other people. I have leaflets printed, which I present to each case that comes to my office. It is much better that he should know he has the disease and that other people should not suffer in consequence.

With regard to the duty of the state, to me it seems to be a matter which it should grasp with all its power, and I want to enter an appeal here and if possible have this association take action, which may be presented to the state legislature to the effect that we approve of state control of tubercular cases, the establishment of sanitaria for the treatment of incipient cases, as well as hospitals for the care of more advanced cases who should be prevented spreading the disease. We confine the insane lest they kill or injure others. Why should we not confine tubercular patients who will not do as they ought, but submit everybody else to the dread danger of consumption?

Another point, and it applies as well to cancer, is that the state should prevent the marriage of tubercular patients. Even when the patient has so far recovered as to show no evidence of the disease he should not be allowed to marry. It is a matter of some disturbance to the individual, of course, but personal feelings should not be allowed to enter into the question.

Dr. H. R. Gaylord—I really do not know what I can say on this subject, my connection with the state laboratory being of a purely scientific character, and, scientists, as a rule, are peculiarly sceptical on the subject of statistics. I think it almost impossible to prove anything of importance from them. From a clinical standpoint they are of considerable value. My personal opinion with regard to tuberculosis is, that one of the very first things the state should attack is the cattle question. From a scientific standpoint it bears a very marked relation to the spread of tuberculosis. I think in regard to its taking systematic care of patients, that there would result a very marked difference in statistics now based entirely on laboratory work.

With regard to cancer, I can appreciate what has been said about our work and can understand the attitude taken in regard to the statistics. About it I express no personal opinion. I can say that there is throughout the scientific world a great deal of conservative scepticism regarding carcinoma, which is entirely justifiable. I came to Buffalo a sceptic, having been trained in a laboratory where very little thought was given to parasitic theory, but I stand today on a somewhat different footing. My experience has caused me to change a great many of my scientific views. It would not be wise for me to make any statements with regard to the work carried on in the laboratory, but I can say that they are infinitely more surprising to me now than they will be to you when published. I am satisfied that in a few years, perhaps months, we shall have something definite to say about cancer.

Dr. W. H. Bergtold—It seems to me if I apprehend the situation, that the chief use of Dr. Pryor's paper is to teach us not to go off at half-cock. I think that statistics may be manipulated unconsciously, if you please, and that applies to my own paper and in fact covers almost any position. It seems to me that in regard to this question of tuberculosis and cancer, we are hoping and expecting a Utopia in ten or fifteen years. You are not going to make a race moral in ten years, nor are you going to educate it in ten, nor fifty, nor one hundred years. If we simply call attention to the fact that tuberculosis is infectious we are teaching only half the subject; we must teach them that there must be a soil, that we must take care of that soil, to make it unresponsive to the seed. Any man who fails to teach his patient to take care of all his excretions is criminally negligent; and he who does not teach his patient to build up his bodily resistance is equally so. I think if we could carry out Dr. Rochester's suggestion it would be wise. However, the doctor tacitly assumes that all people are virtuous and that the prevention of marriage would prevent propagation. If that were true, it would be well, but the prevention of marriage would not by any means stop the coming into the world of people with the hereditary diathesis.

Dr. Pryor—Said he had believed for a long time that we cannot depend upon statistics, but to take the ground that all statistics are inaccurate is simply absurd. If there be one thing a man can learn to figure on absolutely it is vital statistics. The error lies not in the figures but in the conclusions drawn from them. The position he took was simply that he did not believe certain statistics nor the conclusions based thereon.

